# The Prevalence of Tick-Borne Encephalitis Virus in the Ticks and Humans of China from 2000 to 2023: A Systematic Review and Meta-Analysis

**DOI:** 10.3390/vetsci12020146

**Published:** 2025-02-08

**Authors:** Hongyu Qin, Xiu Xin, Qichao Tang, Xujing Feng, Baishuang Yin

**Affiliations:** 1 Key Lab of Preventive Veterinary Medicine in Jilin Province, Jilin 132101, China; qinhyvet@163.com (H.Q.); xxvet1988@163.com (X.X.); qichaotang1988@163.com (Q.T.); xiujingfengvet@163.com (X.F.); 2 College of Animal Science and Technology, Jilin Agricultural Science and Technology University, Jilin 132101, China

**Keywords:** ticks, tick-borne encephalitis, pooled prevalence, epidemiology, China

## Abstract

This study presents a systematic review and meta-analysis of studies on tick-borne encephalitis virus (TBEV) prevalence in ticks and human hosts in China, published between 2000 and 2023. Extensive searches were conducted on four databases—PubMed, CNKI, VIP, and Wan Fang. The findings indicated overall pooled prevalence estimates of TBEV infection in ticks and humans of 5.8% and 9.0%, respectively. The prevalence of TBEV in ticks was 4.8% (95%CI, 4.5–7.1%) during 2000–2010 and increased to 6.3% (95%CI, 4.7–7.8%) during 2011–2023, and was mainly distributed in Jilin (13.4%; 95%CI, 8.3–18.4%) and Inner Mongolia (4.5%; 95%CI, 1.8–7.1%). The prevalence of TBEV was higher in *Dermacentor silvarum* than in other tick species (8.1%; 95%CI, 3.2–12.9%). The seroprevalence of TBEV in humans increased from 4.7% (95%CI, 2.9–6.4%) during 2000–2010 to 17.6% (95%CI, 11.3–23.8%) during 2011–2023. Simultaneously, the analysis results of the population characteristics showed that females, forestry workers, military personnel, and farmers were found to be susceptible, and the highest seroprevalence was noted among homemakers and unemployed individuals. There were certain differences in the seroprevalence among populations in different provinces, especially in Xinjiang and Heilongjiang. This study can provide a reference for a more comprehensive and in-depth investigation of ticks and humans infected with TBEV in China.

## Simple Summary

Ticks are important vectors of many pathogens, including tick-borne encephalitis virus (TBEV), which can cause severe neurological damage in humans and animals. This study examined information gathered from several sources, such as research publications, to evaluate the overall impact of TBEV in China. The overall pooled prevalence in China was calculated to be 5.8% in ticks and 9.0% in humans. The results showed differences in TBEV prevalence between different regions, tick species, and periods. Moreover, the investigation found other factors that influence the spread of TBEV, including geographical factors and occupations. In summary, the review of the literature provided significant information about the occurrence and distribution of TBEV in China. This study improves understanding of the epidemiological characteristics of TBEV and aids in formulating prevention and control strategies in China.

## 1. Introduction

Approximately 70% of the emerging infectious diseases on a global scale can be attributed to zoonotic ailments, characterized by their transmission pathway from animals to humans [1,2]. Ticks were the first arthropods to be recognized as vectors capable of transmitting pathogens to humans and worldwide, and as vectors of infectious diseases, they are second only to mosquitoes [3]. Tick-borne infections are zoonoses and their pathogens are maintained in natural cycles that involve tick vectors and animal hosts. Humans are occasional hosts for ticks and are generally regarded as dead-end hosts, having no role in maintaining tick-borne agents in nature [4]. Different tick species show preferences for various biotopes or environments and these preferences determine their geographical distribution and, as a result, the areas at risk of human tick-borne infections [5]. Over the past three decades, tick-borne pathogens have emerged across the globe and currently pose a significant threat to human and animal health [6].

Ticks are a type of obligate blood-sucking parasite from three Arthropod families: Ixodidae, Nuttalliellidae, and Argasidae [7]. More than 800 tick species have been identified worldwide, among which the Ixodidae has the largest number of species, exceeding 700. To our knowledge, there are more than 120 species of ticks—including over 110 species in the Ixodidae (so-called hard tick) family and 19 species in the Argasidae (soft tick) family—in China, which are widely distributed across various geographical and ecological environments [8,9]. Geographical environment, vegetation, and climate affect the geographical distribution of ticks, and the pathogens carried by ticks are often specific to the tick species. In China, a suite of tick-borne diseases (TBD) have been documented, with notable prevalence among the human population. Prominent among these are Lyme disease, tick-borne encephalitis, Crimean–Congo hemorrhagic fever, Q fever, tularemia, North Asia tick-borne spotted fever, Human Granulocytic Anaplasmosis (HGA), and Severe Fever with Thrombocytopenia Syndrome [10]. Consequently, an augmented focus has been bestowed upon ticks and the array of diseases they transmit across the expanse of China. Nevertheless, the significance of tick-associated pathogens and the resultant diseases remains undervalued within the Chinese context [11].

Tick-borne encephalitis virus (TBEV) is one of the tick-borne viruses infecting humans. It belongs to the Flaviviridae family and causes central nervous system lesions in humans and animals. Once a tick is infected, it will carry TBEV throughout its life and be permanently infectious [12,13]. The virus is widely prevalent in Eurasia, and as many as 13,000 cases of tick-borne encephalitis are reported every year [14,15]. Based on the geographical locations of early sample sources, as well as serological and phylogenetic data, the subtypes of TBEV recognized by the International Committee on Taxonomy of Viruses (ICTV) mainly comprise three types, namely the European subtype (Eu-TBEV), the Far Eastern subtype (FE-TBEV), and the Siberian subtype (Sib-TBEV) [16]. In recent years, due to an increase in sample size and more in-depth research, two new subtypes, “178–179” and “886–884”, have been proposed based on genomic differences. The former is only represented by the 178–179 strain, while the latter, also known as the Baikalian subtype (Bkl-TBEV), includes 886–884 and multiple related strains [17,18]. In addition, the Himalayan subtype (Him-TBEV) has been isolated from the wild Himalayan marmot in the Qinghai–Tibet Plateau in China [19].

The TBEV isolates in China mainly belong to the TBEV-FE subtype and are widely distributed across multiple provinces, including Heilongjiang, Jilin, Inner Mongolia, Xinjiang, Yunnan, and Tibet, while the TBEV-Sib subtype has only been reported in Xinjiang. In China, TBEV is mainly transmitted through the bites of *Ixodes persulcatus*, and there are genetic differences between TBEV isolates in different natural foci [19,20]. The Far Eastern subtype (TBEV-FE) is mainly prevalent in China, and researchers detected the Siberian subtype (TBEV-Sib) of TBEV in the Xinjiang Uygur Autonomous Region for the first time in 2016 [21].

Ticks play an important role in TBEV transmission and pose a challenge to public health security. In China, patients with tick-borne encephalitis virus are mainly distributed in Northeast China, such as Heilongjiang Province, Yunnan Province, and the Xinjiang Uygur Autonomous Region, and are mainly infected with the Far East Asian type. In Northeast China, the main vector of tick-borne encephalitis is *Ixodes persulcatus*, while in the Tianshan Mountains and Alatau Mountains of Xinjiang, the main vector of TBEV is *Dermacentor silvarum*. Relevant studies in Yunnan show that the main vector is *Ixodes ovatus*. There is a strong correlation between several subtypes of tick-borne encephalitis virus, so there are similarities in their prevention and control.

The risk of ticks transmitting TBEV to humans is increasing due to economic development and urbanization intensifying worldwide. It is expected that two-thirds of the population will live in cities within 30 years. During the urbanization process, people’s activities of land reclamation and the exploitation of forest resources increase the probability of contact with ticks. Due to these multi-factor changes, the contact between ticks and humans is increasing, and TBE has begun to show new epidemic trends and characteristics on a global scale. In this study, the prevalence of TBEV in tick and human populations in China was investigated through a meta-analysis, and the factors related to its infection were evaluated and analyzed. The results from this analysis will not only aid in the effective control of tick-borne encephalitis, but also improve public health safety.

## 2. Materials and Methods

### 2.1. Search Strategy

The “Preferred Reporting Items for Systematic Reviews and Meta-Analyses statement (PRISMA)” protocols were followed during this meta-analysis [22]. We performed a systematic search across four electronic databases—the China National Knowledge Infrastructure (CNKI), the Wiper Chinese Journal Database (VIP), the Wan Fang database (WF), and the PubMed database—using the following Mesh terms and keyword subject headings: “Tick”, “Tick-borne Encephalitis”, “Forest Encephalitis”, “Tick-borne Encephalitis Virus”, “Forest Encephalitis Virus”, “Serology”, and “seroprevalence”. When searching PubMed, the keyword “China” was added. We focused on studies with positive results on TBEV in ticks and human serum TBEV in China. Only studies conducted from 1 January 2000 to 25 December 2023 were included, to reflect the current data.

### 2.2. Inclusion Criteria

Existing reviews, duplicate publications, and studies and reports that only reported the serum positive rate without original data were excluded. Additionally, studies with unclear descriptions of diagnostic methods or species were also excluded from this analysis. Studies were included if they met the following conditions: (1) the study used ticks or the general population in China; (2) the study reported the positive rate of TBEV in ticks; (3) the content involved the seroprevalence of TBEV antibodies in human serum; (4) the test method and processes were clearly stated; and (5) the content included the source of the research object and the characteristics of the population. If the results were inconsistent, they were resolved by a third party or through negotiation and discussion. Articles that did not meet the above criteria were excluded.

### 2.3. Literature Screening and Data Extraction

Reviewers carried out the extraction and recording of data from each chosen study independently. In case of any discrepancies between the reviewers or any ambiguity regarding the suitability of a study, additional reviewers were employed to resolve the issue. Information was recorded as follows: The data extracted regarding the prevalence of TBEV included the first author, year of publication, year of sample isolation, province, detection method, number of tick samples, number of positives, and tick species. The data extracted regarding the prevalence of TBEV antibodies in human serum covered gender, age, and occupation. No attempt was made to contact the authors of the original studies for Supplementary Information, nor did we locate any unpublished data. Microsoft Excel 2019 was used for data management.

### 2.4. Quality Assessment

The quality of the eligible literature was assessed based on the standard derived from the Grading of Recommendations Assessment, Development, and Evaluation Method (GRADE) framework [23,24]. The quality of the publications was graded using a scoring approach. In brief, for each of the subsequent items, a score of 1 point was assigned if the relevant information was provided in detail: the subject of the study, the detection approach employed, the sampling year, and the subgroup classification. Two independent researchers carried out data collection and quality evaluation, and any differences were settled through mutual agreement. Papers were assigned 0–4 points based on a pre-defined score criterion. Studies with 3–4 points were considered high quality, those with 2 points were deemed moderate, and those with scores of 0–1 point were considered to be of low quality.

### 2.5. Statistical Analysis

The prevalence of TBEV in ticks and the seroprevalence of TBEV in human serum were calculated by meta-analysis of all the included publications. Heterogeneity was expected in the qualified studies and was estimated using the I^2^ test, following which the effect model was selected [25,26]. A random-effects model (REM) was selected as the pooling method if significant heterogeneity among studies was observed (*p* < 0.1 and I^2^ > 50%). The source of heterogeneity was analyzed through a meta-regression [27]. Otherwise, a fixed-effect model (FEM) was used [28]. All effective quantities were expressed as 95% confidence intervals (CI), and *p* < 0.05 defined statistical significance.

A funnel plot was employed to evaluate the presence of publication bias among the included studies. The funnel plot displayed notable asymmetry, suggesting significant publication bias. Egger’s test is commonly used to assess potential publication bias via funnel plot asymmetry, where *p* ≥ 0.05 indicates a low risk of publication bias, while *p* < 0.05 suggests possible bias [29,30]. To assess the consistency and stability of the meta-analysis, a sensitivity analysis was performed. This involved systematically excluding one study at a time and recalculating the combined prevalence of TBEV risk [30]. Additionally, subgroup and meta-regression analyses were conducted to evaluate potential sources of heterogeneity and identify factors contributing to the observed heterogeneity. When analyzing the total prevalence, subgroups were compared based on sample collection time (before 2010 (2000–2009) and those in 2010 or later (2010–2023)), administrative districts or regions, tick species, and human genders and occupations. The data were analyzed using Stata software (version 15.0). All *p* values were two-sided, and *p* values < 0.05 were considered significant.

## 3. Results

### 3.1. Studies Included

**Figure 1:**
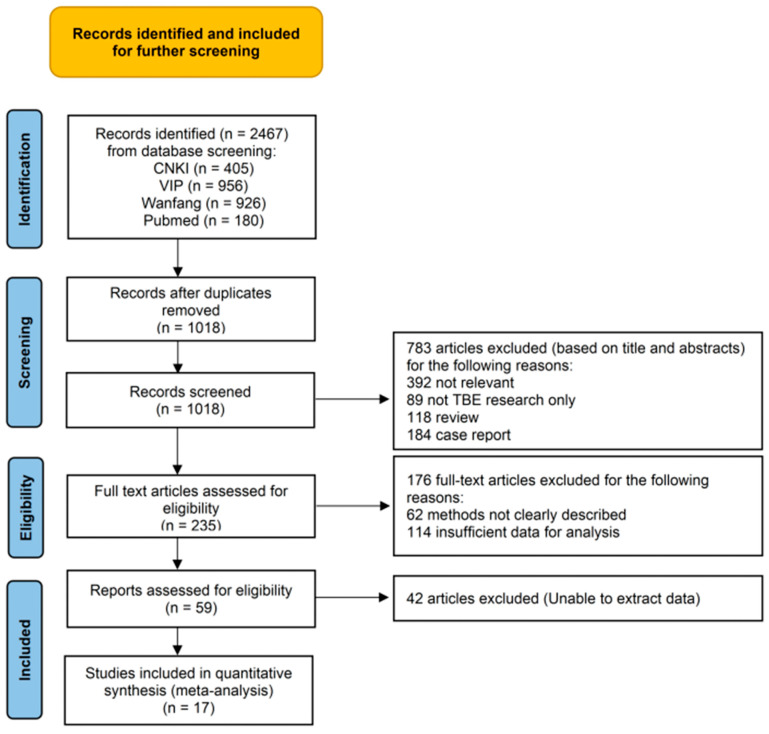
Flow diagram of reference screening regarding prevalence of TBEV in ticks.

A total of 2467 relevant articles related to the prevalence of TBEV in ticks were retrieved, and 1018 studies were chosen after preliminary screening and the elimination of repeated works. Another 959 studies were further excluded due to discrepancies in the research content, and 42 studies were excluded because the prevalence data provided were insufficient for extracting complete data. The remaining 17 studies were ultimately included in the meta-analysis (Table S1). A flow diagram of the specific literature search is shown in Figure 1.

**Figure 2:**
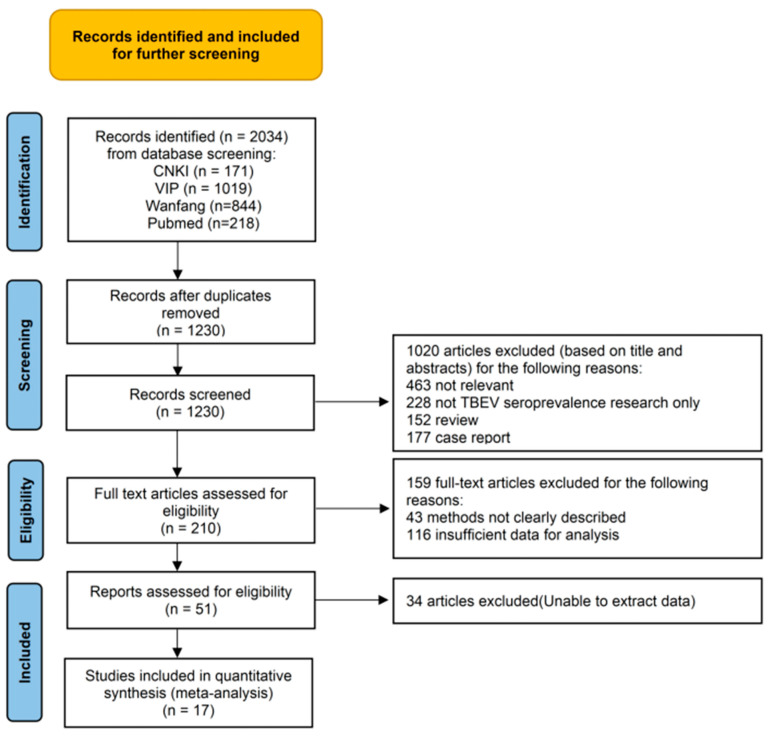
Flow diagram of reference screening regarding prevalence of TBEV seroprevalence in human serum.

Also, a total of 2034 articles related to the seroprevalence of TBEV in human serum in China were retrieved from the four databases, and after initial screening and removing duplicates, 1230 studies were chosen for analysis. From these, 1179 studies were excluded due to inconsistent research content, and 34 studies were excluded because of insufficient sample data. Finally, a quantitative analysis was conducted on the 17 remaining articles (Table S2). The specific literature retrieval process is shown in Figure 2.

### 3.2. Heterogeneity Analysis

**Figure 3:**
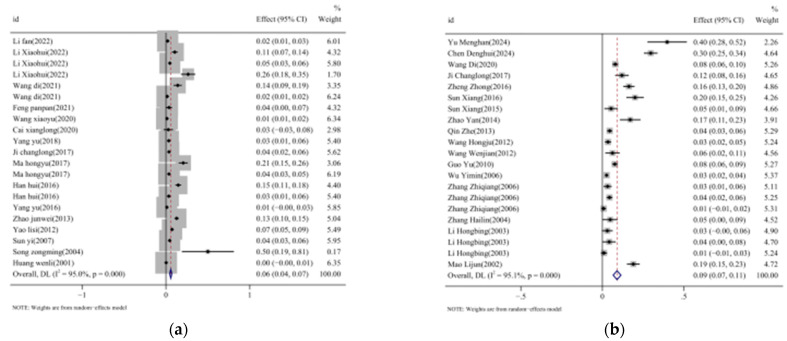
Summary forest plot of TBE incidence from random-effects analyses. Note: (**a**) studies on TBEV prevalence in ticks [20,31,32,33,34,35,36,37,38,39,40,41,42,43,44,45,46]; (**b**) studies on TBEV seroprevalence in human serum [37,47,48,49,50,51,52,53,54,55,56,57,58,59,60,61,62].

We used double arcsine conversion on the prevalence data of TBEV in ticks and seroprevalence data in human serum from the included studies to estimate their 95% confidence intervals. Heterogeneity tests were conducted on all studies included in the final analysis, yielding I^2^ values of 95.0% (TBEV in ticks) and 95.1% (seroprevalence in humans), which indicates significant heterogeneity (I^2^ > 50%) in the literature selected for this study. A random-effects model was used for the analysis, which combined the incidence rates from each study. As shown in Figure 3a,b, the results show the pooled prevalence estimate of TBEV in ticks (5.8% [95%CI: 4.5–7.1%]) and the seroprevalence of TBEV in humans (9.0% [95%CI: 6.6–11.3%]) in different regions of China.

### 3.3. Publication Bias and Sensitivity Analysis Bias

**Figure 4:**
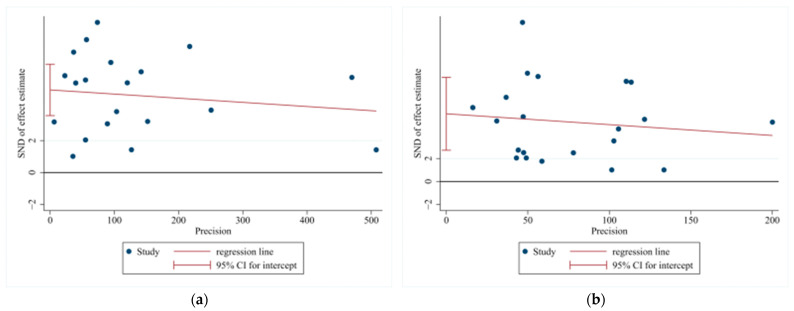
Egger test of publication bias among TBEV studies. Note: (**a**) studies on TBEV prevalence in ticks; (**b**) studies on TBEV seroprevalence in human serum.

**Figure 5:**
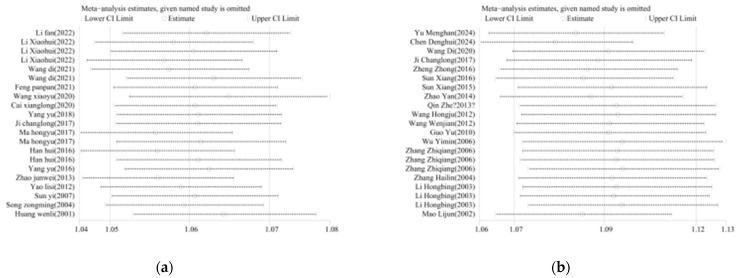
Sensitivity analysis of TBEV incidence. Egger test of publication bias among studies. Note: (**a**) studies on TBEV prevalence in ticks [20,31,32,33,34,35,36,37,38,39,40,41,42,43,44,45,46]; (**b**) studies on TBEV seroprevalence in human serum [37,47,48,49,50,51,52,53,54,55,56,57,58,59,60,61,62].

Egger’s test was employed to assess potential publication bias, and the result was *p* = 0.001 < 0.05, suggesting a significant risk of publication bias, as presented in Figure 4a,b. In light of the considerable heterogeneity detected by the meta-analysis of TBEV incidence rates, a sensitivity analysis was carried out on the incorporated literature. By successively excluding individual studies during the sensitivity analysis [63], it was found that the effect sizes invariably resided within the 95% confidence interval of the final effect size. This attests to the satisfactory stability and robustness of the results, as illustrated in Figure 5a,b.

### 3.4. Subgroup Analysis of Prevalence of TBEV in Ticks

**Figure 6:**
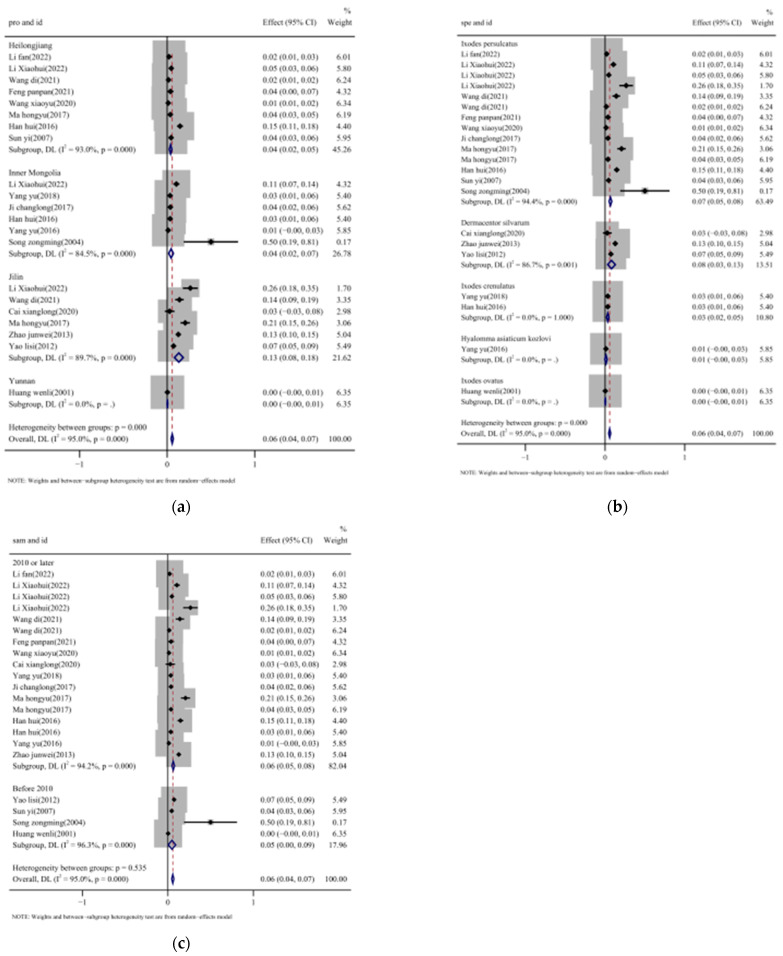
Forest plot of estimated TBEV prevalence in ticks, estimated with random-effects subgroup analyses. Note: (**a**) subgroup analysis of different provinces [20,31,32,33,34,35,36,37,38,39,40,41,42,43,44,45,46]; (**b**) subgroup analysis of different tick species [20,31,32,33,34,35,36,37,38,39,40,41,42,43,44,45,46]; (**c**) subgroup analysis of sample times [20,31,32,33,34,35,36,37,38,39,40,41,42,43,44,45,46].

In order to explore the origin of the heterogeneity, a subgroup analysis was conducted, with the corresponding results detailed in Table 1 and Figure 6. The province subgroup analysis revealed that the prevalence of TBEV in ticks was 13.4% (95%CI: 8.3–18.4%) in Jilin, followed by 4.5% (95% CI: 1.8–7.1%) in Inner Mongolia, and 3.9% (95%CI: 2.5–5.4%) in Heilongjiang, while Yunnan had the lowest prevalence (Figure 6a). There were significant differences (*p* = 0.000) in prevalence between provinces.

The tick species subgroup analysis demonstrated that *Dermacentor silvarum* exhibited the highest prevalence at 8.1% (95%CI: 3.2–12.9%), followed by *Ixodes persulcatus* at 6.7% (95%CI: 5.0–8.5%), *Dermacentor nuttalli* at 3.4% (95%CI: 1.9–5.0%), *Hyalomma asiaticum kozlovi* at 1.1% (95%CI: −0.4–2.7%), and *Ixodes ovatus* with the lowest prevalence at 0.3% (95%CI: −0.1–0.7%) (Figure 6b).

The prevalence of TBEV in ticks has been increasing gradually over the past 20 years. It was 4.8% (95%CI: 0.4–9.2%) during 2000 and 2010. Later, between 2011 and 2023, the prevalence reached 6.3% (95%CI: 4.7–7.8%), demonstrating the significant yet gradual increase over the past 20 years in TBEV prevalence in ticks in China (Figure 6c).

### 3.5. Subgroup Analysis of Seroprevalence of TBEV in Humans in China

**Figure 7:**
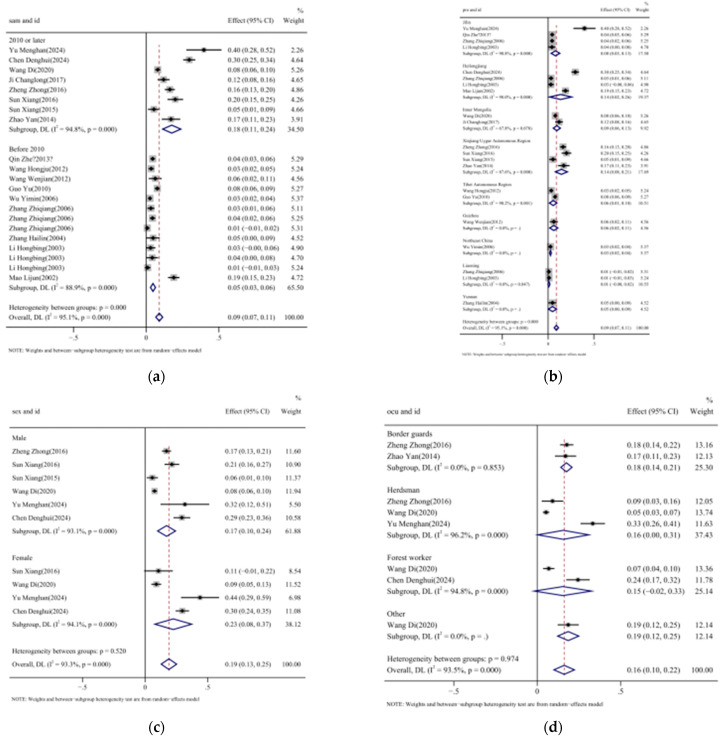
Forest plot of TBEV seroprevalence in human serum, estimated with random-effects subgroup analyses. Note: (**a**) subgroup analysis of sample times [37,47,48,49,50,51,52,53,54,55,56,57,58,59,60,61,62]; (**b**) subgroup analysis of different provinces [37,47,48,49,50,51,52,53,54,55,56,57,58,59,60,61,62]; (**c**) subgroup analysis by gender [47,48,49,50,51,52]; (**d**) subgroup analysis of different occupations [47,48,49,50,53].

We aimed to evaluate TBEV’s distribution and seroprevalence in humans in China. According to the analysis results (Table 2), the seroprevalence of TBEV in humans was 4.7% (95%CI: 2.9–6.4%) from 2000 to 2010, and 17.6% (95%CI: 11.3–23.8%) between 2011 and 2023. The prevalence has been increasing gradually over the past 20 years. The details are shown in Figure 7a.

Our analysis demonstrates that, at the province level, the seroprevalence in humans was the highest in Xinjiang Uygur Autonomous Region (14.5%, 95%CI: 7.9–21.1%), followed by Heilongjiang, Inner Mongolia, Jilin, Guizhou, and Tibet Autonomous Region (13.6%, 9.5%, 8.0%, 6.2%, 5.5%, respectively; Figure 7b).

No significant differences in prevalence were observed for gender and occupation (*p* = 0.520 and 0.974, respectively). In terms of gender distribution, the seroprevalence was 17.2% in males (95%CI: 10–24.3%) and 22.5% in females (95%CI: 7.8–37.2%) (Figure 7c). The results of the occupational analysis demonstrated that the seroprevalence of TBEV was 17.5% in border guards (95%CI: 14.2–20.9%), 15.7% in herdsmen (95%CI: 4.0–31.0%), 15.4% in forestry workers (95%CI: −1.7–32.6%), and 18.6% in people with other occupations (95%CI: 12.3–25.0%) (Figure 7d).

## 4. Discussion

Tick-borne encephalitis is mainly caused by the bites of ticks carrying TBEV, which can invade the central nervous system and cause severe neurological damage. Currently, there is no specific treatment for this disease, and active immunization is the main preventive measure [64,65]. Tick-borne encephalitis virus belongs to the Flavivirus genus in the Flaviviridae family and has a genome of approximately 11 kb [66]. It is widely distributed in Europe and Asia and is prevalent in 27 European countries and at least 4 Asian countries. It is considered a human health threat, infecting 10,000–12,000 humans worldwide each year [67,68,69,70]. It has been included in the list of notifiable infectious diseases in the European Union since 2012 and is under routine surveillance [71]. The tick-borne encephalitis virus and other tick-borne viruses are also continuously detected through tick surveillance in Europe, the United States, and the Netherlands [72,73,74,75].

In China, tick-borne encephalitis is one of the occupational diseases caused by biological factors listed in the statutory occupational disease catalog, and it is classified as a non-statutory notifiable infectious disease. However, only Heilongjiang Province has designated it as a statutory notifiable infectious disease within the province, although its disease burden is still unclear [76,77]. The actual number of people infected with TBE is considered higher than the reported number of cases. In recent years, the number of TBE infection cases in European and Asian countries, including China, has continued to increase [78,79]. Yet, despite the increasing number of reported cases of tick-borne encephalitis virus infections in the Chinese Mainland, no comprehensive assessments of this major public health issue have been conducted. This new epidemic trend of TBE is a serious threat to public health in the Eurasian region and a thorough meta-analysis of cases is crucial to grasp the current incidence characteristics of TBE and gain a detailed understanding of the epidemiological features of tick-borne encephalitis in China. This is of great significance for the prevention and control of tick-borne virus infections in China.

This systematic review and meta-analysis clearly shows changes in the distribution of TBE in both ticks and the human population over the past two decades, scientifically analyzing the changes in the epidemic characteristics of TBE. We demonstrate that the prevalence of TBEV in *Dermacentor silvarum* is higher than in *Ixodes persulcatus*, which is different from previous research results, indicating that the tick species transmitting TBE in China has changed. The distribution characteristics and population abundance of ticks are closely related to their ecological environment [76,80]. The lower prevalence of the TBE virus in *Ixodes persulcatus*, despite its larger population compared to *Dermacentor silvarum,* can be attributed to multiple factors. Ecological changes caused by human activities, such as deforestation and reforestation, have modified forest landscapes across different regions [81]. These alterations may have created a more favorable environment for *Dermacentor silvarum*, favoring its survival and proliferation over *Ixodes persulcatus*. Simultaneously, changes in host availability and behavior are also influential. If the main hosts of *Dermacentor silvarum* become more abundant or change their distribution patterns, this tick has a higher likelihood of contracting and transmitting the TBE virus compared to *Ixodes persulcatus*. A broader or altered host range can increase the tick’s exposure to the virus. Moreover, the migratory patterns of host animals play a role [82]. Migratory hosts may spread the virus to new areas, enhancing the infection risk of *Dermacentor silvarum* and leading to a higher prevalence of the virus it carries. In contrast, *Ixodes persulcatus* may be less exposed to the virus due to its specific ecological requirements, its host preferences, and the timing of its interactions with hosts. However, further research is essential to comprehensively understand and confirm these potential causes and their complex interactions, which is crucial for better assessing and managing the risk of TBE transmission in China.

Our meta-analysis revealed that there are significant differences in TBE epidemic monitoring in different regions of China. For Northeast China, the epidemic monitoring mainly focuses on TBE positivity in ticks, and there is little published research on human serum antibody positivity. This may be because Northeast China has always been a high-TBE incidence area, and a good prevention and treatment protocol has been established in the population, with relatively high vaccination awareness. Despite few human cases of tick-borne encephalitis virus (TBEV), high rates of TBEV seroprevalence have been reported among humans and animals in Xinjiang Uygur Autonomous Region in Northwestern China [83]. Meanwhile, the TBE epidemic situation in Northwest China is relatively complex due to the less typical TBEV (Siberian and Far Eastern subtypes of TBE coexist) transmission, with multiple confusing tick-borne viruses, complex host animal situations, and a research focus on assessing threats to human health—along with limited detection resources, more attention is paid to detecting human serum antibody positivity. The development in different areas of Xinjiang is unbalanced, with some areas being less informed about TBE. According to the existing studies, the antibody seroconversion rate of Xinjiang herdsmen is relatively high after the TBE epidemic season, and the number of patients is very small [21]. Despite this, there are many cases of latent infection, and it is still necessary to actively carry out serological monitoring, promote vaccination, and monitor local epidemic strains. With the development of national support policies and ecological tourism, the number of foreign tourists in Xinjiang has increased, which brings additional challenges to the control of TBE. Considering this, it is important to actively publicize TBE prevention and control, clearly outline the TBE epidemic areas and TBE prevalence in ticks, and establish an effective prevention, diagnosis, and treatment system in China.

Recent studies have shown that herdsmen, homemakers, and the unemployed have become the main affected population, while the number of infected forestry workers and border guards has remained stable, which is consistent with the results of this study. The high TBEV seropositivity rate among homemakers and the unemployed, in addition to the traditionally recognized groups like military personnel, herdsmen, and forestry workers, can be attributed to several factors. The expansion of human activities into tick-infested areas is a significant contributor. Homemakers who engage in activities such as gathering wild plants or mushrooms in forested regions near their homes, or who accompany family members on outdoor trips, are increasingly exposed to ticks. Their activities often involve more in-depth exploration of tick-prone areas compared to some of their other family members. Additionally, they may lack sufficient awareness of tick-borne disease prevention, making them more vulnerable to tick bites. The unemployed, who might have more unstructured time and potentially engage in subsistence activities or hobbies in natural environments, also face a higher risk [84]. In addition, changes in land use and the encroachment of human settlements into previously wild habitats have led to a closer proximity between ticks and a broader range of the population [8]. This means that individuals who were previously less likely to encounter ticks are now more frequently in contact with them. Moreover, the lack of awareness and preventive measures among these groups compared to professional forestry workers or military personnel trained in tick-borne disease prevention plays a role. Without proper knowledge of tick avoidance and protection, they are more vulnerable to tick bites and subsequent TBEV infection. At present, the vaccination of forestry workers in this area should continue, and the immunization coverage of all groups of people entering the forest area should be increased. Education regarding TBE in high-incidence areas should be increased, and local farmers and homemakers should be vaccinated. Additionally, the presence of TBE in ticks in high-incidence areas should be monitored to detect changes in TBE positivity rates, tick species distribution, regional distribution, and epidemic strain changes early, and to provide scientific data for controlling and reducing the incidence in the population.

The present study has several limitations that should be acknowledged. One key limitation is the uneven geographic coverage of the included studies, which were predominantly focused on the northern regions of China. The studies did not provide comprehensive representation across all provinces, cities, and autonomous regions within China. This geographic bias may limit the generalizability of the findings, as tick distribution and abundance can vary substantially across different ecological regions within the country. Future research should aim to expand the geographic scope and ensure more balanced sampling across China’s diverse environments to obtain a more comprehensive understanding of tick population dynamics. Another limitation is the potential for publication bias, which may have influenced the overall conclusions of this study.

The uneven geographic coverage of the included studies could lead to an overestimation of the TBEV infection rate, as certain regions may have higher prevalence. Additionally, the usefulness of the data in informing public health policy is constrained by the lack of comprehensive information on the overall clinical burden and severity of TEBV infection. The current findings may not accurately represent the true variations in risk across China’s diverse regions and populations.

Future research should aim to address these limitations by expanding the geographic scope, ensuring more balanced sampling, and collecting robust data on the clinical epidemiology of TBEV. This would provide a more accurate and representative assessment that can better guide public health decision-making and intervention strategies. Despite these caveats, the present findings can still contribute valuable information to the understanding of tick-borne TBEV infections in China.

## 5. Conclusions

This meta-analysis provides a comprehensive overview of the prevalence of TBEV in tick and human populations in China from 2000–2023. The results show significant heterogeneity in the prevalence data, which is influenced by various factors such as geographical region, tick species, and period. Changes in population infection rates and the differences in TBEV prevalence among different regions highlight the need for targeted prevention and control strategies. Vaccination remains a key preventive measure, and efforts should be made to expand vaccination coverage to other high-risk groups beyond forestry workers. Continued surveillance of TBEV in both tick and human populations is essential for monitoring the epidemic trend and adapting prevention and control measures accordingly, thus safeguarding public health from the threat of tick-borne encephalitis.

Overall, this meta-analysis provides valuable insights into the epidemiological patterns of TBEV in China, which can guide the development of more targeted and effective public health interventions to mitigate the burden of tick-borne encephalitis in the country.

## Figures and Tables

**Table 1:** Frequency of TBEV prevalence in ticks, and subgroup analysis.

Subgroup	Variables	No. Studies	No. Samples	No. Positive	Prevalence % (95%CI)	Heterogeneity		
						Z	*p*	I^2^
Species	*Dermacentor silvarum*	5	1680	181	8.1 (3.2–12.9)	4.317	0.000	0
	*Ixodes persulcatus*	14	8528	338	6.7 (5.0–8.5)	7.614	0.000	94.40%
	*Dermacentor nuttalli*	2	484	17	3.4 (1.9–5.0)	1.422	0.155	0
	*Hyalomma asiaticum kozlovi*	1	178	2	1.1 (−0.4–2.7)	3.260	0.001	86.70%
	*Ixodes ovatus*	1	717	2	0.3 (−0.1–0.7)	1.416	0.157	0
Sampling	2011–2023	13	9523	454	6.3 (4.7–7.8)	7.757	0.000	94.20%
	2000–2010	4	2184	89	4.8 (0.4–9.2)	2.151	0.031	96.30%
Province	Jilin	6	1753	221	13.4 (8.3–18.4)	3.304	0.000	84.50%
	Inner Mongolia	6	1379	69	4.5 (1.8–7.1)	5.177	0.000	89.70%
	Heilongjiang	8	7858	251	3.9 (2.5–5.4)	5.238	0.000	93.00%
	Yunnan	1	717	2	0.3 (−0.1–0.7)	1.416	0.000	0

**Table 2:** Frequency of TBEV seroprevalence in human serum, and subgroup analysis.

Subgroup	Variables	No. Studies	No. Samples	No. Positive	Prevalence % (95%CI)	Heterogeneity		
						Z	*p*	I^2^
Sampling	2011–2023	8	2533	400	17.6 (11.3–23.8)	5.506	0.000	94.80%
	2000–2010	13	4549	253	4.7 (2.9–6.4)	25.256	0.031	88.90%
Province	Xinjiang	4	894	142	14.5 (7.9–21.1)	4.297	0.000	87.60%
	Heilongjiang	4	1124	216	13.6 (1.6–25.6)	2.225	0.000	98.00%
	Inner Mongolia	2	1120	98	9.5 (5.6–13.4)	4.784	0.078	67.80%
	Jilin	4	1250	77	8.0 (3.3–12.8)	3.314	0.000	90.80%
	Tibet	2	1256	81	5.5 (1.4–9.6)	2.647	0.001	90.20%
	Guizhou	1	113	7	6.2 (1.8–10.6)	2.732	0.000	0
	Liaoning	2	234	2	0.8 (−0.3–0.2)	1.407	0.847	0.00%
	Yunnan	1	84	4	4.8 (0.2–9.3)	2.049	0.000	0
Gender	Male	6	1584	227	17.2 (10.0–24.3)	4.739	0.000	92.90%
	Female	4	549	122	22.5 (7.8–37.2)	3.006	0.003	94.10%
Occupation	Border guard	2	490	86	17.5 (14.2–20.9)	10.212	0.000	0
	Herdsman	3	760	88	15.7 (0.4–31.0)	2.008	0.045	96.20%
	Forest worker	2	359	50	15.4 (−1.7–32.6)	1.761	0.078	94.80%
	Other	1	145	27	18.6 (12.3–25.0)	5.158	0.000	0

## Data Availability

The data are contained within the article and Supplementary Materials.

## Supplementary Materials

The following supporting information can be downloaded at: https://www.mdpi.com/article/10.3390/vetsci12020146/s1: PRISMA checklist; Table S1, Included studies of TBEV prevalence in ticks in China; Table S2, Included studies of TBEV seroprevalence in human serum in China.
