# Supplementary material for: The Prevalence of Tick-Borne Encephalitis Virus in the Ticks and Humans of China from 2000 to 2023: A Systematic Review and Meta-Analysis
Source: Vet Sci. 2025 Feb 8;12(2):146. doi: 10.3390/vetsci12020146 (PMC11861706; doi:10.3390/vetsci12020146)
Supplement: Supplementary file 1 [file vetsci-12-00146-s001.zip › vetsci-3415142-supplementary.pdf]

Supplementary Material 1: PRISMA checklist

SECTION	ITEM	PRISMA-ScR CHECKLIST ITEM	REPORTED ON PAGE #
**TITLE**			
Title	1	Identify the report as a scoping review.	**Title**
**ABSTRACT**			**ABSTRACT**
Structured summary	2	Provide a structured summary that includes (as applicable): background, objectives, eligibility criteria, sources of evidence, charting methods, results, and conclusions that relate to the review questions and objectives.	**Abstract**
**INTRODUCTION**			**INTRODUCTION**
Rationale	3	Describe the rationale for the review in the context of what is already known. Explain why the review questions/objectives lend themselves to a scoping review approach.	**Introduction, Paragraph 1-4**
Objectives	4	Provide an explicit statement of the questions and objectives being addressed with reference to their key elements (e.g., population or participants, concepts, and context) or other relevant key elements used to conceptualize the review questions and/or objectives.	**Introduction, Paragraph 5-6**
**METHODS**			**METHODS**
Protocol and registration	5	Indicate whether a review protocol exists; state if and where it can be accessed (e.g., a Web address); and if available, provide registration information, including the registration number.	**Methods, Paragraph 1-6**

Eligibility criteria	6	Specify characteristics of the sources of evidence used as eligibility criteria (e.g., years considered, language, and publication status), and provide a rationale.	**Methods, Paragraph 2**
Information sources*	7	Describe all information sources in the search (e.g., databases with dates of coverage and contact with authors to identify additional sources), as well as the date the most recent search was executed.	**Methods, Paragraph 1**
Search	8	Present the full electronic search strategy for at least 1 database, including any limits used, such that it could be repeated.	**Methods, Paragraph 1**
Selection of sources of evidence†	9	State the process for selecting sources of evidence (i.e., screening and eligibility) included in the scoping review.	**Methods, Paragraph 2**
Data charting process‡	10	Describe the methods of charting data from the included sources of evidence (e.g., calibrated forms or forms that have been tested by the team before their use, and whether data charting was done independently or in duplicate) and any processes for obtaining and confirming data from investigators.	**Methods, Paragraph 2**
Data items	11	List and define all variables for which data were sought and any assumptions and simplifications made.	**Methods, Paragraph 3**
Critical appraisal of individual sources of evidence§	12	If done, provide a rationale for conducting a critical appraisal of included sources of evidence; describe the methods used and how this information was used in any data synthesis (if appropriate).	**Methods, Paragraph 4**
Synthesis of results	13	Describe the methods of handling and summarizing the data that were charted.	**Methods, Paragraph 5-6**

**RESULTS**			**RESULTS**
Selection of sources of evidence	14	Give numbers of sources of evidence screened, assessed for eligibility, and included in the review, with reasons for exclusions at each stage, ideally using a flow diagram.	**Results, paragraph 1-2 Figure1-2**
Characteristics of sources of evidence	15	For each source of evidence, present characteristics for which data were charted and provide the citations.	**Results, paragraph 1-2 Supplementary Table1-2**
Critical appraisal within sources of evidence	16	If done, present data on critical appraisal of included sources of evidence (see item 12).	**Results, paragraph 3-4 Figure 3-5**
Results of individual sources of evidence	17	For each included source of evidence, present the relevant data that were charted that relate to the review questions and objectives.	**Results, paragraph 8-10 Figure 6-7 Table 3-4**
Synthesis of results	18	Summarize and/or present the charting results as they relate to the review questions and objectives.	**Results, paragraph 8-10 Figure 6-7 Table 3-4**
**DISCUSSION**			**DISCUSSION**
Summary of evidence	19	Summarize the main results (including an overview of concepts, themes, and types of evidence available), link to the review questions and objectives, and consider the relevance to key groups.	**Discussion, paragraph 1-5**
Limitations	20	Discuss the limitations of the scoping review process.	**Discussion, paragraph 6-8**

Conclusions	21	Provide a general interpretation of the results with respect to the review questions and objectives, as well as potential implications and/or next steps.	**Conclusions**
**FUNDING**			**FUNDING**
Funding	22	Describe sources of funding for the included sources of evidence, as well as sources of funding for the scoping review. Describe the role of the funders of the scoping review.	**Funding**

Table S1. Included studies of TBEV prevalence in ticks in China

Study ID	Province/region	Region	Species	Sampling years	No. tested	No. positive	Detection method	Study design	Quality score
Li F.(2022)[1]	Heilongjiang	Northeast China	Ixodes persulcatus	2010 or later	475	10	PCR	Cross sectional	3
Li X. (2022)[2]	Inner Mongolia	Northeast China	Ixodes persulcatus	2010 or later	285	30	PCR	Cross sectional	4
Li X. (2022)	Heilongjiang	Northeast China	Ixodes persulcatus	2010 or later	643	30	PCR	Cross sectional	4
Li X. (2022)	Jilin	Northeast China	Ixodes persulcatus	2010 or later	102	27	PCR	Cross sectional	4
Wang D.(2021)[3]	Jilin	Northeast China	Ixodes persulcatus	2010 or later	192	27	PCR	Cross sectional	4
Wang D.(2021)	Heilongjiang	Northeast China	Ixodes persulcatus	2010 or later	963	15	PCR	Cross sectional	4
Feng P.P.(2021)[4]	Heilongjiang	Northeast China	Ixodes persulcatus	2010 or later	108	4	PCR	Cross sectional	4
Wang X.Y.(2020)[5]	Heilongjiang	Northeast China	Ixodes persulcatus	2010 or later	2761	35	PCR	Cross sectional	3
Cai X.L.(2020)[6]	Jilin	Northeast China	Dermacentor silvarum	2010 or later	35	1	PCR	Cross sectional	4
Yang Y.(2018)[7]	Inner Mongolia	Northeast China	Dermacentor nuttalli	2010 or later	263	9	PCR	Cross sectional	4
Ji C.L.(2017)[8]	Inner Mongolia	Northeast China	Ixodes persulcatus	2010 or later	260	11	PCR	Cross sectional	4
Ji C.L.(2017)	Inner Mongolia	Northeast China	Ixodes crenulatus	2010 or later	120	3			
Ma H.Y.(2017)[9]	Jilin	Northeast China	Ixodes persulcatus	2010 or later	218	45	PCR	Cross sectional	4
Ma H.Y.(2017)	Heilongjiang	Northeast China	Ixodes persulcatus	2010 or later	1652	60	PCR	Cross sectional	4

Han H.(2016)[10]	Heilongjiang	Northeast China	Ixodes persulcatus	2010 or later	5	1	PCR	Cross sectional	4
Han H.(2016)	Heilongjiang	Northeast China	Dermacentor silvarum	2010 or later	397	58			
Han H.(2016)[11]	Inner Mongolia	Northeast China	Dermacentor nuttalli	2010 or later	221	8	PCR	Cross sectional	4
Han H.(2016)	Inner Mongolia	Northeast China	Dermacentor silvarum	2010 or later	42	1			
Yang Y.(2016)[12]	Inner Mongolia	North China	Hyalomma asiaticum kozlovi	2010 or later	178	2	PCR	Cross sectional	4
Zhao J.W.(2013)[13]	Jilin	Northeast China	Dermacentor silvarum	2010 or later	603	77	PCR	Cross sectional	4
Yao L.S.(2012)[14]	Jilin	Northeast China	Dermacentor silvarum	Before 2010	603	44	PCR	Cross sectional	4
Sun Y.(2007)[15]	Heilongjiang	Northeast China	Ixodes persulcatus	Before 2010	854	38	PCR	Cross sectional	4
Song Z.M.(2004)[16]	Inner Mongolia	Northeast China	Ixodes persulcatus	Before 2010	10	5	animal experiment	Cross sectional	2
Huang W.L.(2001)[17]	Yunnan	Southwest China	Ixodes ovatus	Before 2010	717	2	/	Cross sectional	2

Table S2. Included studies of TBEV seroprevalence in human serum in China

Study ID	Province/region	Region	Sampling years	No. tested	No. positive	Detection method	Study design	Quality score
Yu M.H.(2024)[18]	Jilin	Northeast China	2010 or later	63	25	IFA	Cross sectional	4
Chen D.H.(2024)[19]	Heilongjiang	Northeast China	2010 or later	456	135	PCR	Cross sectional	4
Wang D.(2020)[20]	Inner Mongolia	Northeast China	2010 or later	886	70	PCR	Cross sectional	4
Ji C.L.(2017)[8]	Inner Mongolia	Northeast China	2010 or later	234	28	IFA	Cross sectional	1
Zheng Z.(2016)[21]	Xinjiang Uygur Autonomous Region	Northwest China	2010 or later	431	70	IFA	Cross sectional	3
Sun X.(2016)[22]	Xinjiang Uygur Autonomous Region	Northwest China	2010 or later	215	43	IFA	Cross sectional	3
Sun X.(2015)[23]	Xinjiang Uygur Autonomous Region	Northwest China	2010 or later	113	6	IFA	Cross sectional	4
Zhao Y.(2014)[24]	Xinjiang Uygur Autonomous Region	Northwest China	2010 or later	135	23	IFA	Cross sectional	3
Qin Z.（2013）[25]	Jilin	Northeast China	Before 2010	629	28	IFA	Cross sectional	3
Wang H.J.(2012)[26]	Tibet Autonomous Region	Southwest China	Before 2010	350	12	IFA	Cross sectional	3
Wang W.J.(2012)[27]	Guizhou	Southwest China	Before 2010	113	7	IgM	Cross sectional	2
Guo Y.(2010)[28]	Tibet Autonomous Region	Southwest China	Before 2010	906	69	IFA	Cross sectional	2
Wu Y.M.(2006)[29]	Northeast China	Northeast China	Before 2010	1007	26	IFA	Cross sectional	1

Zhang Z.Q.(2006)[30]	Heilongjiang	Northeast China	Before 2010	188	6	IFA	Cross sectional	2
Zhang Z.Q.(2006)	Jilin	Northeast China	Before 2010	462	20	IFA	Cross sectional	2
Zhang Z.Q.(2006)	Liaoning	Northeast China	Before 2010	133	1	IFA	Cross sectional	2
Zhang H.L.(2004)[31]	Yunnan	Southwest China	Before 2010	84	4	HI	Cross sectional	1
Li H.B.(2003)[32]	Heilongjiang	Northeast China	Before 2010	100	3	IFA	Cross sectional	2
Li H.B.(2003)	Jilin	Northeast China	Before 2010	96	4	IFA	Cross sectional	2
Li H.B.(2003)	Liaoning	Northeast China	Before 2010	101	1	IFA	Cross sectional	2
Mao L.J.(2002)[33]	Heilongjiang	Northeast China	Before 2010	380	72	IgM	Cross sectional	2

References

1. LI, F.; LIU, F.M.; FENG, P.P.; WANG, D.Q.; FU, S.H.; NIE, K. Investigation of tick-borne viruses in Huanan county，Heilongjiang province，China，**2020**. Chin J Vector Biol & Control 2022, 33, 637-641.
2. Li, X.; Ji, H.; Wang, D.; Che, L.; Zhang, L.; Li, L.; Yin, Q.; Liu, Q.; Wei, F.; Wang, Z. Molecular detection and phylogenetic analysis of tick-borne encephalitis virus in ticks in northeastern China. Journal of medical virology **2022**, 94, 507-513.
3. Wang, D.; Ji, H.W.; Wang, Z.D.; Li, X.H.; Zhang, L.; Wei, F.; Liu, Q. Isolation and identification of forest encephalitis virus carried by ticks in parts ofNortheast China. cHIN J Vet Sci **2021**, 41, 469-474.
4. FENG, P.P. Investigation of Tick-borne viruses in parts of the Sino-Russian border. Master's Thesis, Chinese Center For Disease Control and Prevention, **2021**.
5. WANG, X.Y.; MA, Y.J.; SUN, Z.D.; SUO, J.N.; WANG, X.; JIAO, Y.; ZHANG, S.T.; CHEN, X. Investigation on distribution of ticks and carrying status of tick borneencephalitis virus in Heilongiiang Province. Chin J of PHM **2020**, 36, 750-752.
6. CAI, X.L.; YAN, X.M.; DONG, X.; HE, B.; ZHU, Y. Detection and genetic characterization of tick-borne flavivirus in parts of Heilongjiang and Jilin provinces. Chinese

Journal of Preventive Veterinary Medicine **2020**, 42, 1116-1122.
7. YANG, Y.; GAO, Y.; CAO, Y.; WANG, J.; DENG, H.; WANG, J.; CHEN, X.; LIU, L.; XU, B. Investigation on tick-borne pathogens in Inner Mongolia Manchuriaport area during 2012-2014. Chin J Vector Biol & Control **2018**, 29, 147-150.
8. JI, C.L. Investigation on the foci of tick-borne forest encephalitis in Alatanheli area of Bogda Mountain. Cardiovascular Disease Journal of integrated traditional Chinese and Western Medicine **2017**, 5, 82-83.
9. MA, H.Y. Discovery and identification ofNew Viruses in Ticksfrom Northeastern China. Master's Thesis, Jilin Agricultural University, **2017**.
10. HAN, H.; WU, H.L.; HU, X.F.; SONG, Y.J.; XU, B.L. Surveillance on tick-borne pathogens at Heilongjiang port. Chinese Frontier Health Ouarantine **2016**, 39, 413-416.
11. HAN, H.; YANG, Y.; ZHAO, X.; SONG, Y.J.; XU, B.L. Monitoring for tick and tick-borne pathogensat Inner Mongolia ports. Acta Parasitol. Med. Entomol. Sin. 2016, 23, 224-229.
12. YANG, Y.; JingWANG; WANG, J.; ZHANG, S.; LI, H.; LI, Y.; HAO, G.; MA, Z.; WANG, W.; XU, B. Investigation on tick-borne pathogens in Ganqimaodu port areas onthe borders between China and Mongolia,2012-2013. Chinese Frontier Health Ouarantine **2016**, 39, 330-332+347.
13. ZHAO, J.W. Tick-borne pathogent investigation in Jilin and Heilongjiang Province. Master's Thesis, Chinese Center For Disease Control and Prevention, **2013**.
14. YAO, L.S. Study of Composition of rodents andtheir ectoparasites and.Pathogens Infection in the adjacent port area ofChangbaimountain of China and Democratic People's Republic of Korea(DPRK). Doctoral Thesis, Academy of Military Medical Sciences, **2012**.
15. SUN, Y.; LIU, G.P.; YANG, L.W.; XU, R.M.; YU, Y.X. Multiple infections of tick-borne pathogens in Ixodes persulcatus collected from forests in Heilongjiang province. aCTA Parasitol Med Entomol Sin **2007**, 231-240.
16. Zongming, S.; Liang, H.; Chenglong, H.; Hongli, Z.; Dacheng, Y.; Hua, L.; Liancheng, Z.; Shuangjun, L.; Liguang, Y. Studies on epidemic disease of tick-borne encephalitis(TBE) surveillancen and TBE virus isolated. Letters in Biotechnology **2004**, 15, 43-44+50.
17. HUANG, W.L.; HOU, Z.L.; ZI, D.Y.; GONG, Z.D.; LEI, Y.M.; MI, Z.Q.; ZHANG, H.L. Investigation of the Russian SpringSummer Encephalitis Virus in Yunnan Province. Chinese Journal of Preventive Veterinary Medicine **2001**, 23, 72-74.
18. YU, M.H. Study on clinical nucleic acid testing and serological testing of tick-borne encephalitis virus infection. Master's Thesis, Jilin University, **2024**.
19. Chen, D.; Lu, Y.; Wang, W.; Zhang, Y.; Liu, T.; Liu, H.; Zhang, L.; Peng, X.; Lv, S.; Wang, Z. The Prevalence of Tick-Borne Encephalitis Virus Infection Among Humans in Heilongjiang Province of China in 2020-2023. Zoonoses and public health **2024**, 71, 955-961.
20. WANG, D. Epidemiological investigation and analysis of tick borne encephalitis virus(TBEV) in Northeast China. Master's Thesis, Jilin Agricultural University, **2020**.
21. ZHENG, Z.; ZHANG, G.; ZENG, F.; SUN, X.; LIU, R.; LIU, X.; JIA, J. Seroepidemiological Investigation on Human Infections withTick-borne Encephalitis in Xinjiang. J Prev Med Chin PLA **2016**, 34, 806-808.

22. SUN, X.; ZHANG, G.L.; ZHENG, Z.; LIU, X.M.; LIU, R.; QIU, E.; ZHAO, Y. Serological Investigation on Tick-borne Diseases in forest staff in Northern Xinjiang. Acta Parasitol. Med. Entomol. Sin. **2016**, 23, 158-163.
23. SUN, X.; ZHANG, G.L.; LIU, R.; LIU, X.M.; YANG, L.L.; ZHENG, Z.; ZHAO, Y. Tick-borne encephalitis endemic foci in Altai Mountains, Xinjiang, China. Chinese Journal of Zoonoses **2015**, 31, 1189-1192.
24. ZHAO, Y.; LIU, R.; ZHANG, G.L.; LIU, X.M.; SUN, X.; ZHENG, Z.; QIU, E.C. Seroepidemiological investigation of tick-borne diseases in a border guard unit in northern Xinjiang. J Prev Med Chin PLA **2014**, 32, 324-325.
25. QIN, Z.; FENG, L.; QIN, D.Z.; YU, M.; WANG, Z.; ZHANG, Z.Q.; WU, Y.M. A serological study on tick borne infectious diseases amongresidents in Changbai mountainous area. Chin Prev Med **2013**, 14, 886-888.
26. WANG, H.J.; HU, S.L.; NI, Z.; ZHUO, M.Y.; WANG, Q.Z.; LI, S.L.; WU, D.; ZHANG, X.; LI, X.J.; BU, D.; et al. Serosurvey for four arthropod-borne infectious diseases in Linzhi. South China J Prev Med **2012**, 38, 16-20.
27. WANG, W.J.; AN, M.H.; YOU, Y.G.; AN, N.O.; LI, J.Q.; YE, X.F.; ZHAO, S.Y.; LIU, C.T. Surveillance report of viral encephalitis in Dejiang County, Guizhou Province from 2007 to 2009. Applied Preventive Medicine **2012**, 18, 241-243.
28. GUO, Y. Seroepidemiological investigation of forest encephalitis in Milin, Tibet. Chinese and Foreign Health Abstracts **2010**, 9-10.
29. WU, Y.M.; ZHANG, Z.Q.; WANG, H.J.; GUAN, G.K.; FENG, L.; WANG, L.Q.; HU, L.M. Serological survey of tick-borne infectious diseases in some areas of Northeast China. J Prev Med Chin PLA **2006**, 24, 300.
30. ZHANG, Z.Q.; WU, Y.M.; FENG, L.; WANG, H.J.; WANG, L.Q.; LIU, G.P.; CAI, Z.L. Investigation of forest encephalitis virus antibody in some areas of northeast China. Shenyang Army medicine **2006**, 19, 112-113.
31. ZHANG, H.L.; ZHANG, Y.Z.; YANG, W.H.; ZHANG, Y.Z.; MI, Z.Q.; HUANG, W.L.; YUAN, Q.H.; WANG, J.L. Investigation on the Antibodies Aqainst Arboviruses in Sera of Hman being and Ani main the Lower Reaches Area of lancang River in Yunnan Province. Journal of Medical Pest Control **2004**, 20, 207-211.
32. LI, H.B.; HU, L.M.; WEI, A.M.; ZHANG, Z.Q.; LIU, H.; LU, Z.X. Investigation of several tick-borne infectious disease antibodies in some areas of three provinces in Northeast China. Shenyang Budui Yiyao **2003**, 16, 371-372.
33. MAO, L.J.; ZHAO, J.Y.; XU, X.X.; SUN, S.Y. Clinical analysis on 380 cases of forest encephalitis. Chinese J Ind Med **2002**, 15, 137-140.
